# Somatostatin Receptor Type 2 as a Potential Marker of Local Myocardial Inflammation in Myocardial Infarction: Morphologic Data on Distribution in Infarcted and Normal Human Myocardium

**DOI:** 10.3390/biomedicines12102178

**Published:** 2024-09-25

**Authors:** Vyacheslav V. Ryabov, Andrey A. Trusov, Maria A. Kercheva, Aleksandra E. Gombozhapova, Julia N. Ilyushenkova, Ivan V. Stepanov, Mikhail V. Fadeev, Anna G. Syrkina, Svetlana I. Sazonova

**Affiliations:** 1 Department of Emergency Cardiology, Cardiology Research Institute, Tomsk National Research Medical Center, Russian Academy of Sciences, Tomsk 634012, Russia; rvvt@cardio-tomsk.ru (V.V.R.); mariiakercheva@mail.ru (M.A.K.); gombozhapova@gmail.com (A.E.G.); sag@cardio-tomsk.ru (A.G.S.); 2 Nuclear Medicine Department, Cardiology Research Institute, Tomsk National Research Medical Center, Russian Academy of Sciences, Tomsk 634012, Russia; ilyushenkova_cardio@mail.ru (J.N.I.); sazonova_si@mail.ru (S.I.S.); 3 Department of Pathology, Cardiology Research Institute, Tomsk National Research Medical Center, Russian Academy of Sciences, Tomsk 634012, Russia; i_v_stepanov@mail.ru (I.V.S.); fadeev_m_v@mail.ru (M.V.F.)

**Keywords:** myocardial infarction, inflammation, SSTR, SSTR-targeted radioimaging, macrophages, neutrophils, pericytes, molecular imaging, PET, SPECT

## Abstract

Nuclear imaging modalities can detect somatostatin receptor type 2 (SSTR2) in vivo as a potential marker of local post-MI inflammation. SSTR2+ macrophages are thought to be the main substrate for SSTR-targeted radioimaging. However, the distribution of SSTR2+ cells in the MI patients’ myocardium is unknown. Using immunohistochemistry, we investigated the distribution of SSTR2+ cells in the myocardium of patients who died during the MI inflammatory phase (n = 7) compared to the control group of individuals with fatal trauma (n = 3). Inflammatory cellular landscapes evolve in a wave front-like pattern, so we divided the myocardium into histological zones: the infarct core (IC), the border zone (BZ), the remote zone (RZ), and the peri-scar zone (PSZ). The number of SSTR2+ neutrophils (NPs), SSTR2+ monocytes/macrophages (Mos/MPs), and SSTR2+ vessels were counted. In the myocardium of the control group, SSTR2+ NPs and SSTR2+ Mos/MPs were occasional, SSTR2+ vessels were absent. In the RZ, the picture was similar to the control group, but there was a lower number of SSTR2+ Mos/MPs in the RZ. In the PSZ, SSTR2+ vessel numbers were highest in the myocardium. In the IC, the median number of SSTR2+ NPs was 200 times higher compared to the RZ or control group myocardium, which may explain the selective uptake of SSTR-targeted radiotracers in the MI area during the inflammatory phase of MI.

## 1. Introduction

To date, it is widely accepted that inflammation is an essential component of cardiovascular disease (CVD) and myocardial infarction (MI) pathogenesis [1,2]. Acute myocardial ischemic injury triggers both a local inflammatory reaction and generalized activation of the immune system [3,4,5,6]. Bone marrow and the spleen are major sources of leukocytes that fuel inflammation in the ischemic myocardium [7,8,9]. A well-balanced inflammatory response is necessary for the favorable resolution of MI. In light of the above, individual characteristics of the inflammatory response are thought to influence clinical outcomes in MI patients [2,3,4,6,10]. In infarcted myocardium, the number of specific immune cell populations and their phenotypes change rapidly during the healing process [6,11,12,13]. Cardiac repair in MI has the following phases: the early phase (first 24 h), the inflammatory phase (~2–6 days in humans), the proliferative phase (~7–14 days), and the maturation phase (~from 2 weeks to months) [7,14,15].

In recent years, immunomodulatory drugs have demonstrated effectiveness in patients with CVDs [16,17,18]. However, the downsides of anti-inflammatory therapy are infectious complications and the potential suppression of myocardial regeneration when the patient’s inflammatory response is already reduced. In order to achieve an optimal risk–benefit ratio, it is necessary to develop personalized criteria for prescribing immunomodulatory therapy, as well as diagnostic methods to identify these criteria in clinical practice.

But, new challenges are emerging on the path to personalized immunomodulatory therapies for MI:

Insufficient understanding of the mechanisms of the local immune response to ischemic cardiac injury in vivo in humans. Most of the data have been obtained in animal models or cell cultures;

The criteria for distinguishing a physiological immune response to ischemic injury from a pathological one are unclear;

To date, there is no method for monitoring local cardiac post-MI inflammation that has been validated for use in clinical practice.

In view of the above, a topical issue in cardiology is the search for markers and methods for non-invasive monitoring of local post-MI cardiac inflammation. Data on local processes in the infarcted myocardium in vivo are essential in order to develop methods of managing post-MI healing. In recent years, somatostatin receptor (SSTR) type 2 (SSTR2) has been extensively studied as a marker for the non-invasive detection and monitoring of local cardiac inflammation, using nuclear imaging modalities [19,20,21,22,23,24,25].

### 1.1. Somatostatin and Its Receptors

Somatostatin (SST), also known as somatotropin release inhibitory factor (SRIF), is a cyclopeptide that exerts antiproliferative, antisecretory, anti-inflammatory and antinociceptive effects; in particular, SST acts on immune and endothelial cells [26,27,28,29,30].

The SRIF system comprises seven genes encoding two peptide precursors (SST/SRIF, cortistatin), and five somatostatin receptors (SSTR1-5) [27]. SST is produced in a multitude of cells and organs. It has been identified in the central nervous system, the hypothalamus, the gastrointestinal tract, the endocrine pancreas, numerous tumors, the thymus, the spleen, bone marrow, and, notably, the heart. Activated immune cells, endothelial cells, synovial cells, and fibroblasts are able to release SST [26,28,29]. SST plays a mediator role in the migration of immune cells (including neutrophils (NPs)) towards sites of inflammation and their release of pro-inflammatory cytokines and mediators [26,28].

SSTRs are G protein-coupled receptors that share common structural features and signaling mechanisms, but differ in their cellular and subcellular localization and mode of regulation [27,31,32]. SSTRs are present in activated immune cells (lymphocytes, monocytes (Mos), macrophages (MPs), dendritic cells), pericytes [33], various neuroendocrine cells, many organs including the brain, the pituitary, the adrenal, the thyroid, the breast, the kidney, the liver, bone marrow, and the spleen [26,27,28,34,35].

### 1.2. SSTR-Targeted Imaging

Octreotide is the most widely used somatostatin analogue, both for treatment and as the basis for SSTR-targeted (SSTR-t) radiotracers. Octreotide is an SSTR2-preferring ligand; it has a high affinity for SSTR2, a moderate affinity for SSTR5, a low affinity for SSTR3, and an extremely low affinity for SSTR1 and SSTR4. Methods for detecting these receptors in vivo are positron emission tomography combined with X-ray computed tomography (PET/CT) or single photon emission computed tomography combined with X-ray computed tomography (SPECT/CT) using SSTR-t radiotracers [27,31,34,36].

SSTR-t imaging was originally developed to detect neuroendocrine tumors, but it was later found that this technique could also be used to detect the foci of acute and chronic inflammation (sarcoidosis, tuberculosis, autoimmune diseases) [25,27,28,37], as well as atherosclerotic plaques in large arteries (coronary arteries, carotid arteries, the aorta) [38,39,40,41]. MPs play a major role in inflammatory processes in the walls of coronary arteries and large vessels. It is hypothesized that MPs are the main contributors to the uptake of SSTR-t radiotracers by the arterial wall [39,41]. The study of post-MI inflammation imaging in clinical practice using SSTR-t radiotracers began in 2015 by Constantin Lapa et al. The results showed a good concordance between MRI findings indicating acute myocardial damage and radiotracer uptake [19]. Tarkin et al. were able to show the uptake of the SSTR-t radiotracer (^68^Ga-DOTATATE), not only in patients immediately after acute MI, but also in patients with chronic ischemic heart disease [20].

Our research group has obtained data demonstrating the feasibility of imaging local post-MI cardiac inflammation using SPECT/CT with the SSTR-t radiotracer 99mTc-Tektrotyd. We hypothesized that more intense myocardial accumulation of 99mTc-Tektrotyd in the subacute stage of MI was related to long-term adverse left ventricular remodeling. According to our data, the intensity (maximum standardized uptake value) of 99mTc-Tektrotyd uptake in the area of recent MI depends directly on the size of ischemic myocardial injury and correlates positively with end diastolic volume and delta end diastolic volume over the 6-month follow-up [21,22,23,24]. However, further larger studies of the clinical and prognostic value of the method and histological validation of nuclear imaging results are needed.

### 1.3. Cellular Expression of SSTRs

The uptake of SSTR-t radiotracers in sites of inflammation has three possible causes [28]: the presence of SSTRs in activated immune cells [26,42], the presence of SSTRs in activated cells of the respective tissue (cardiomyocytes, fibroblasts) [34,43], and SSTRs’ expression by blood or lymphatic vessels (endotheliocytes, pericytes, smooth muscle cells) [44,45,46].

#### 1.3.1. SSTRs in Cardiac Cells

Reverse transcription polymerase chain reaction (RT-PCR) revealed the expression of SSTR1, and SSTR2 mRNA in cardiomyocytes, and the expression of SSTR1, SSTR2, SSTR4, and SSTR5 mRNA in cardiac fibroblasts [43]. At the protein level, cardiomyocytes expressed SSTR2-SSTR5, whereas cardiac fibroblasts showed no significant expression of SSTRs. The latter may be due to the relatively low amount of fibroblasts in the sample [34]. Castillero et al. investigated the cardiac expression of SSTRs in a small cohort of patients with end-stage ischemic cardiomyopathy. Immunohistochemistry (IHC) showed increased SSTR1 and SSTR2 in ischemic cardiomyopathy. The majority of SSTR1 and SSTR2 staining was localized in cardiomyocytes in fibrotic scar-rich areas, without increased inflammatory cell presence [47].

#### 1.3.2. SSTRs in Blood Vessels

Curtis et al. showed that SSTR1 mRNA was always detected in human vessels, whereas the presence of SSTR2 and SSTR4 mRNA was variable, and SSTR3 or SSTR5 mRNA were absent [45]. The same group of authors experimentally demonstrated that SSTR2 was overexpressed at both the mRNA and protein levels in the 1–2 months after balloon catheter injury to the rat iliac artery endothelium [46]. Adams et al. demonstrated the expression of SSTR1 and SSTR3 on inactivated human umbilical vein endothelial cells; SSTR2 expression increased after endothelial cell activation [44].

#### 1.3.3. SSTRs in Immune Cells

In the peripheral blood mononuclear cells and in the spleen, mainly SSTR2 and SSTR3 are found; in Mos, MPs, and in dendritic cells—mainly SSTR2; in B-lymphocytes—mainly SSTR3; in T-lymphocytes—SSTR1-SSTR5, in the thymus—mainly SSTR1, SSTR 2, and SSTR3 [26,42].

Unfortunately, data on somatostatin receptor expression in cardiovascular and immune tissues remain incomplete and contradictory [26,27]. In addition, although we have data on the ability of individual cells to express SSTR2, we do not know which cells express SSTR2 in myocardium during MI and how SSTR2-positive cells are distributed.

### 1.4. Tissue Substrate for SSTR-Targeted Imaging in CVDs

Recently, several studies have been aimed at validating SSTR-t imaging as a technique to detect inflammation in CVDs. One of the objectives of these studies was to identify the cells that contribute most to the uptake of SSTR-t radiotracers by arterial vessels and cardiac tissue.

Boy et al. showed that, in normal human tissue, the uptake of the SSTR-t radiotracer ^68^Ga-DOTATOC was associated with the expression of SSTR2 at the mRNA level [35]. However, this study analyzed mRNA expression in each organ as a whole, so it is unclear which cells within the organ are responsible for radiotracer uptake.

Tarkin et al. performed a comprehensive analysis of the SSTR-t PET tracer ^68^Ga-DOTATATE for imaging atherosclerotic inflammation. The results demonstrated that in atherosclerotic arterial lesions, the substrate for SSTR-t radiotracer accumulation activated M1 MP expressing SSTR2 [41].

Bravo et al. investigated the feasibility of SSTR-t imaging for the detection of myocardial inflammation in sarcoidosis. According to IHC, in three explanted sarcoid hearts and one normal control heart, SSTR2 immunostaining was weakly positive within well-formed granulomas with no significant staining of background myocardium or normal myocardium [48].

Voros et al. provided the first evidence that SST protected cardiomyocytes against ischemia/reperfusion injury. Moreover, SST was expressed in the cardiac tissue at the peptide level, but its mRNA was not detectable. In addition, the authors used the RNA Scope^®^ in situ hybridization method to detect SSTRs in histological sections of the healthy human heart and concluded that SSTR1 and SSTR2 are expressed on vascular endothelial cells and cardiomyocytes. [29]. However, due to the diffuse pattern of RNA in the in situ hybridization signal, data on the co-expression of cell-specific mRNAs with mRNAs of SSTRs should be interpreted with caution.

The accumulated evidence shows the involvement of the SRIF system in MI pathogenesis, and suggests that in addition to diagnostic value, targeting SSTRs may have beneficial therapeutic effects on MI patients [26,27,29,49,50].

In summary, data on SSTR2 expression in peripheral blood leukocytes, atherosclerotic vessels, and sarcoid granulomas led to the hypothesis that MPs are the main substrate for the increased uptake of SSTR-t radiotracers. These findings were extrapolated to post-MI inflammation. In fact, both in atherosclerotic vascular lesions and in sarcoid granulomas, MPs are the main inflammatory cells. However, in MI, the area of ischemic myocardial injury has a much more diverse composition of immune cells. In addition, this complex network of immune cells changes rapidly during MI. But are MPs alone responsible for the uptake of SSTR-t radiotracers in the infarcted heart? To date, there are no direct studies of SSTR2 expression in the myocardium in humans with MI.

Thus, the purpose of this study is to investigate the distribution of different types of SSTR2-positive cells in the myocardium of patients who died in the inflammatory phase of MI compared to the control group.

## 2. Materials and Methods

### 2.1. Ethics

The study was conducted according to the guidelines of the Declaration of Helsinki, and approved by the Biomedical Ethics Committee of the Cardiology Research Institute, Tomsk NRMC (protocol No. 226, dated 24 February 2022). The study was performed in accordance with federal laws and regulations and institutional policies. The post-mortem examination was performed according to Order No. 354n (2013), issued by the Ministry of Health of the Russian Federation. Informed consent for this research was impossible to obtain; therefore, the study was approved by the local Biomedical Ethics Committee. Thus, there was no contradiction to the Declaration of Helsinki (Informed Consent, clause 32).

### 2.2. Patients

A total of 10 patients were included in this study. The main group consisted of 7 patients having fatal type 1 MI with ST segment elevation. Exclusion criteria were MI types 2–5, the presence of infectious complications (sepsis, pneumonia), oncological diseases, valvular heart diseases, and cases where MI was not the primary cause of death. The control group consisted of 3 people (aged from 18 to 55) who died from fatal injuries and were not diagnosed with CVD during their lifetime and had no visible cardiac pathologies according to the autopsy results.

### 2.3. Autopsy

The autopsies were performed within 24 h after the patients’ deaths. At the autopsy, the pathologist macroscopically determined the MI area and collected three fragments of myocardium: (1) a fragment directly from the MI area; (2) a fragment from the border of the infarcted and macroscopically intact myocardium; (3) a fragment of the macroscopically intact myocardium most remote from the MI area.

### 2.4. Tissue Processing

Myocardial samples were fixed for 24 h in 10% neutral buffered formalin. Standard histological processing was carried out using the Excelsior AS Tissue Processor (Thermo Fisher Scientific, Runcorn, UK). The myocardial samples were embedded in paraffin blocks. Microtome sections were made with a thickness of 4 μm and placed on polylysine-coated slides. For a routine pathological examination, histological sections were stained with hematoxylin and eosin, according to standard methods.

### 2.5. Immunohistochemistry

The SSTR2 expression was studied using IHC staining. Rabbit recombinant monoclonal antibodies for the C-terminal fragment of SSTR2 [UMB1] were applied at a dilution of 1/100 (ab134152, Abcam, Cambridge, UK). Langerhans islets of the human pancreas were used as a positive control, while the exocrine part of the pancreas was used as a negative control. IHC staining was performed using a BOND-MAX Fully Automated IHC and ISH Staining System (Leica Biosystems, Melbourne, Australia), according to the following protocol. First, the tissue was deparaffinized and pretreated with antigen retrieval solution (ER1, pH 6.0) at 98 °C for 20 min. After washing, a peroxidase block was applied using a Bond Polymer Refine Detection Kit DS9800 (Leica Biosystems, Nussloch, Germany) for 10 min. The sections were then washed again and incubated with the primary antibody for 30 min and further incubated with the polymer for 10 min. Finally, tissue samples were treated with DAB chromogen for 10 min and counterstained with hematoxylin solution for 10 min.

### 2.6. Scanning, Analysis, Cell Counting

The slides were scanned using an Aperio AT2 microscopeslide scanning system (Leica Biosystems Imaging, Vista, CA, USA). Image viewing, analysis, and cell counting were performed using Aperio ImageScope software (Leica Biosystems Imaging, Vista, CA, USA).

Since myocardial necrosis and the inflammatory cellular landscape evolve in a wave front-like pattern, to show a representative picture, it is necessary to divide the myocardium into histological zones based on their different positions in relation to the focus of ischemia. We identified the following histological zones in the infarcted heart:

(1) Infarct core (IС)—the area with necrotic myocardium;

(2) Border zone (BZ)—the area adjacent to the IC, containing viable cardiomyocytes;

(3) Remote zone (RZ)—the area with intact myocardium, most distant from the IC;

(4) Peri-scar zone (PSZ)—the area around the border where viable cardiomyocytes adjoin scar or mature granulation tissue. We identified this zone because the high concentration of SSTR2+ vessels was found there. This zone was found in two patients (No. 3, No. 6) with reinfarction; they had a combination of a primary MI about 3–8 weeks old and a reinfarction of 3–5 days old, which resulted in death.

In the above zones, the number of SSTR2+ NPs, SSTR2+ Mos/MPs, and SSTR2+ vessels were counted. The identification of the SSTR2+ cell type was based on the characteristic light-optical morphology of the cells. The number of SSTR2-positive cells was counted for each zone in 10 random fields of view (size 600 × 400 μm) with a scale bar of 60 μm. The final number was the arithmetic mean of the number of cells in the 10 fields of view (FOV).

### 2.7. Statistical Analysis

Statistical analysis was performed with the STATISTICA 10.0 software package (StatSoft. Inc., Tulsa, OK, USA). All quantitative indicators that showed abnormal distribution were described by the median (Me) and interquartile interval (Q1; Q3). To test for differences between groups, the Kruskal–Wallis test or the Mann–Whitney U test (Wilcoxon rank-sum test) was used. Results were considered statistically significant if the *p*-value was < 0.05.

## 3. Results

**Figure 1:**
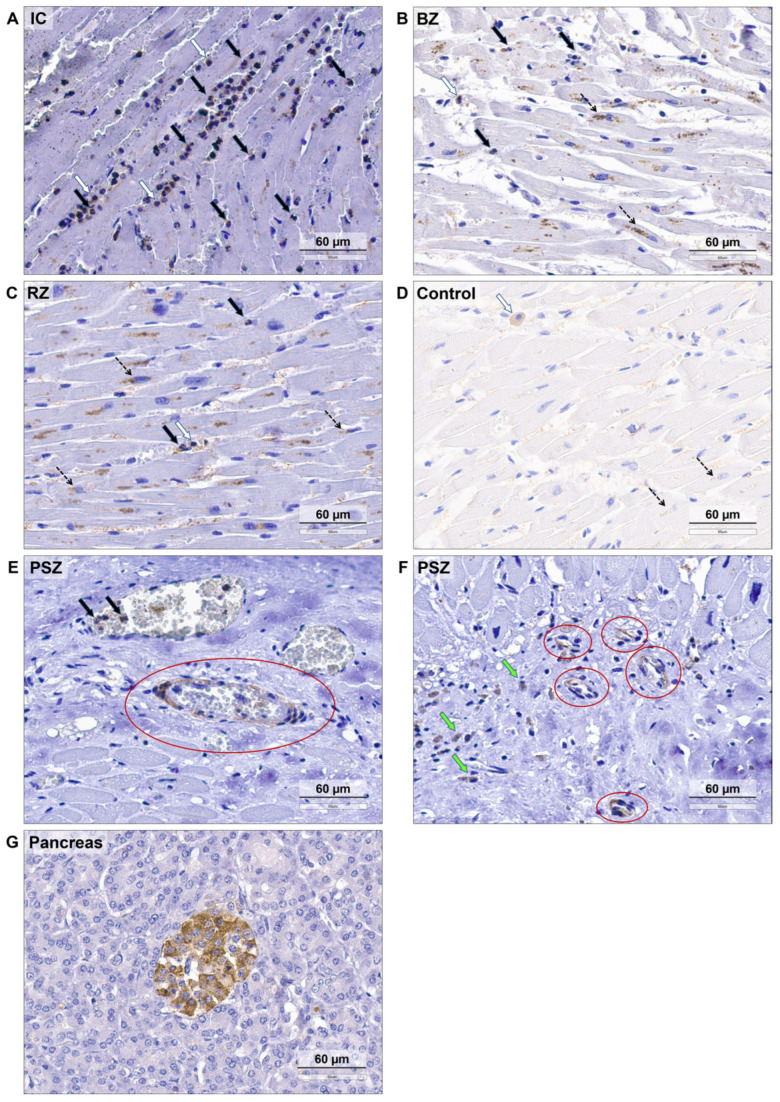
Immunohistochemical images of SSTR2 in myocardial tissue of patients with MI and myocardium of control group. IHC staining with anti-somatostatin receptor subtype-2 antibody (Clone UMB-1; Abcam, Cambridge, MA, USA), work dilution 1:100, counterstained with hematoxylin. Scale bar: 60 μm. (**A**) Infarct core, inflammatory phase (Patient No. 1). Coagulative necrosis of cardiomyocytes with SSTR2+ NP infiltration (brown positive staining, black arrows), in smaller quantities—SSTR2+ Mos (white arrows). (**B**) Border zone, inflammatory phase (Patient No. 1). Viable cardiomyocytes, marked interstitial edema. A small number of SSTR2+ NPs (black arrows), SSTR2+ Mos (white arrow). Perinuclear lipofuscin granules in cardiomyocytes (dashed arrows). (**C**) Remote zone, inflammatory phase (Patient No. 1). Intact cardiomyocytes. There are no SSTR2+ cells in the myocardial tissue itself. Single SSTR2+ NP (black arrows), SSTR2+ Mo (white arrow) in the lumen of the capillaries. Perinuclear lipofuscin granules in cardiomyocytes (dashed arrows). (**D**) Myocardium tissue of the control group. Intact cardiomyocytes with preserved nuclei and transverse strips. Perinuclear lipofuscin granules in cardiomyocytes (dashed arrows). An SSTR2+ MP in the upper left part of the slide (white arrow). (**E**) Peri-scar zone (Patient No. 3). In the upper part of the slide there is mature connective tissue, in the lower part there are hypertrophied cardiomyocytes. In the slide center there is a vessel (red circle) with SSTR2-positive outer layer of cells (pericytes) and SSTR-negative inner layer of cells (endotheliocytes). There are two SSTR-negative vessels above with SSTR2+ NPs in the left vessel lumen (black arrows). (**F**) Peri-scar zone (Patient No. 3). In the lower right part of the slide there is mature connective tissue, in the upper part there are cardiomyocytes. Many SSTR2+ microvessels are visible (red circles). Numerous hemosiderin-laden macrophages with brown granules in cytoplasm are SSTR2-negative (green arrows). (**G**) Pancreas. Langerhans islets of the human pancreas were used as a positive control, while the exocrine part of the pancreas was used as a negative control. IC—infarct core; BZ—border zone; RZ–remote zone; PSZ—peri-scar zone; NP–neutrophil; Mo–monocyte; MP–macrophage.

**Figure 2:**
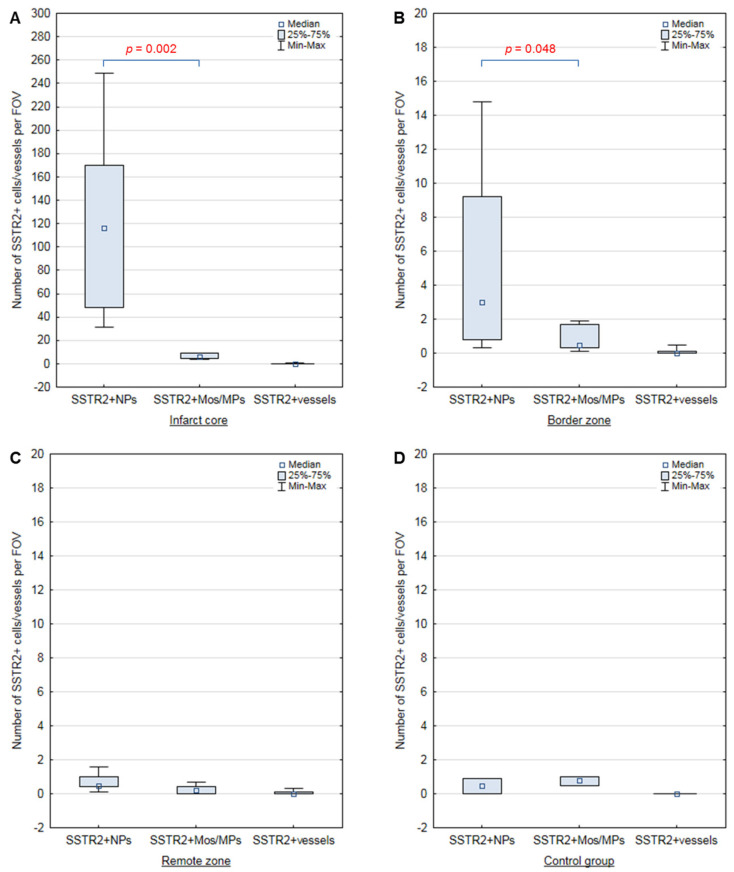
Number of SSTR2+ cells relative to histological zones. (**A**) Infarct core; (**B**) border zone; (**C**) remote zone; (**D**) myocardium of the control group. Data are presented as box plot with median, 25th–75th percentiles (boxes), and minimum-maximum (whiskers). NPs–neutrophils; Mos/MPs–monocytes/macrophages; FOV–field of view.

**Figure 3:**
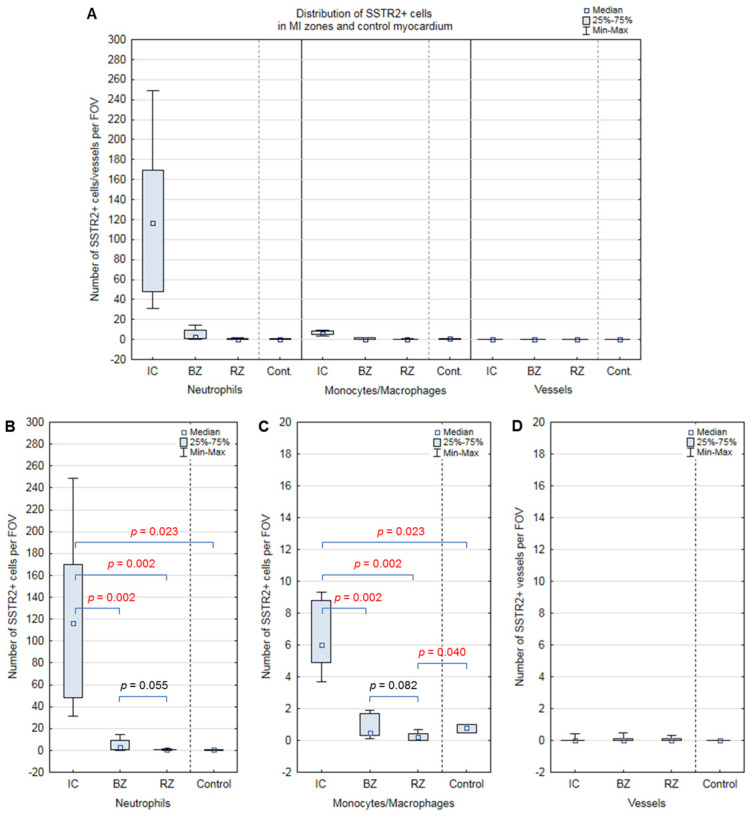
Quantification of SSTR2+ cells in different MI zones in inflammatory phase of MI. (**A**) General picture of SSTR2+ cell distribution in MI zones and control myocardium. (**B**) Distribution of SSTR2+ neutrophils. (**C**) Distribution of SSTR2+ monocytes/macrophages. (**D**) Distribution of SSTR2+ vessels. Data are presented as box plot with median, 25th–75th percentiles (boxes) and minimum-maximum (whiskers). IC—Infarct core; BZ—border zone; RZ—remote zone; FOV—field of view.

The clinical and anamnestic characteristics of the patients are presented in Table 1, Table 2 and Table 3. A typical histologic pattern of SSTR2+ cell distribution for each described zone is shown in Figure 1. The main quantitative data regarding SSTR2+ cells are summarized in Figure 2 and Figure 3, and Table 4.

SSTR2-positive cytoplasmic staining was detected in NPs, some Mos and MPs, some pericytes and endothelial cells, occasional lymphocytes, and occasional epicardial mesothelial cells. In addition, moderate positive extracellular matrix staining was seen in the interstitial spaces between cardiomyocyte fibers. We cannot exclude weak SSTR2-positive staining of cardiomyocytes. However, due to the extremely low staining intensity, it was not possible to distinguish true low-intensity positive staining from non-specific staining. We interpreted this picture as the absence of specific staining in cardiomyocytes.

The majority of SSTR2+ cells are located in the IC. In this zone SSTR2+ NPs predominated, and their numbers were significantly higher than those of SSTR2+ Mos/MPs [SSTR2+ NPs: 116.4 (47.9; 169.8) cells/FOV; SSTR2+ Mos/MPs: 6.0 (4.9; 8.8) cells/FOV; *p* = 0.002]. SSTR2+ vessels were almost absent [0 (0; 0) cells/FOV] (Figure 1A and Figure 2A).

In the BZ, there were significantly more SSTR2+ NPs than SSTR2+ Mos/MPs [SSTR2+ NPs: 3 (0.8; 9.2) cells/FOV; SSTR2+ Mos/MPs: 0.5 (0.3; 1.7) cells/FOV; *p* = 0.048]. Occasional SSTR2+ vessels were found [0 (0; 0.1) cells/FOV] (Figure 1B and Figure 2B).

In the RZ, occasional SSTR2+ NPs [0.5 (0.4; 1) cells/FOV], SSTR2+ Mos/MPs [0.2 (0; 0.4) cells/FOV], and SSTR2+ vessels [0 (0; 0.1) cells/FOV] were found (Figure 1C and Figure 2C).

In the myocardium of the control group, occasional SSTR2+ NPs [0.5 (0; 0.9) cells/FOV] and SSTR2+ Mos/MPs [0.8 (0.5; 1) cells/FOV] were found; SSTR2+ vessels [0 (0; 0) cells/FOV] were absent in the examined FOV (Figure 1D and Figure 2D).

The RZ and control myocardium did not differ in the number of SSTR2+ NPs [RZ: 0.5 (0.4; 1) cells/FOV; control: 0.5 (0; 0.9) cells/FOV; *p* = 0.649] and SSTR2+ vessels [RZ: 0 (0; 0.1) cells/FOV; control: 0 (0; 0) cells/FOV; *p* = 0.569], but the difference in the number of SSTR2+ Mos/MPs was statistically significant [0.2 (0; 0.4) cells/FOV; control: 0.8 (0.5; 1) cells/FOV; *p* = 0.040] (Figure 2C,D).

In the PSZ (Figure 1E,F): SSTR2+ vessels predominated; this zone had the highest concentration of SSTR2+ vessels in the myocardium; the number of SSTR2+ Mos/MPs was about two times higher compared to the control group; the number of SSTR2+ NPs was not clearly different from the control group. However, since this zone was present in only two patients with MI from our sample, it is inappropriate to apply statistical methods; more observations are needed.

SSTR2+ NPs constitute the largest number of SSTR2-positive cells in the infarcted myocardium (Figure 3A). The number of SSTR2+ NPs in the IC [116.4 (47.9; 169.8) cells/FOV] is statistically significantly higher than in the BZ [3 (0.8; 9.2) cells/FOV; *p* = 0.002], RZ [0.5 (0.4; 1) cells/FOV; *p* = 0.002] or the control group [0.5 (0.4; 1) cells/FOV; *p* = 0.023]. The number of SSTR2+ NPs in the BZ is higher compared to the RZ [3 (0.8; 9.2) cells/FOV; 0.5 (0.4; 1) cells/FOV; *p* = 0.055]; however, the P value did not reach the threshold level of statistical significance. We assume that with an increase in the sample, the *p* value will reach the threshold level of significance (Figure 3B).

The number of SSTR2+ Mos/MPs in the IC [6.0 (4.9; 8.8) cells/FOV] is statistically significantly higher than in the BZ [0.5 (0.3; 1.7) cells/FOV; *p* = 0.002], RZ [0.2 (0; 0.4) cells/FOV; *p* = 0.002], or the control group [0.8 (0.5; 1) cells/FOV; *p* = 0.023] (Figure 3C).

The number of SSTR2+ vessels was extremely low and did not differ between IС [0 (0; 0) cells/FOV], BZ [0 (0; 0.1) cells/FOV], RZ [0 (0; 0.1) cells/FOV], and the control group [0 (0; 0) cells/FOV] (Figure 3D).

## 4. Discussion

Different types of SSTR2-positive cells in the myocardium of patients with fatal MI are the most likely substrate for SSTR-t imaging in MI. In most previous studies, researchers have linked the uptake of SSTR-t radiotracers in the inflammatory foci primarily to the presence of activated MPs. However, our data show that not only MPs, but also NPs, Mos and some vessels clearly express SSTR2. Moreover, during the inflammatory phase, SSTR2+ NPs are about 20 times more abundant than SSTR2+ Mos/MPs in the IC, suggesting that in this phase of MI, SSTR-t radiotracer accumulation is mainly due to SSTR2+ NPs. The importance of NPs in the pathogenesis of MI cannot be overstated; in MI, they are both a diagnostic marker and a therapeutic target [51,52,53]. Because NPs have both a destructive potential and pro-reparative functions, a balanced approach is required to improve myocardial healing outcomes [54]. Since the vast majority (maybe all) of the detected NPs express SSTR2, the dynamics of SSTR2+ NPs coincides with the dynamics of the NP response as a whole. In view of the above, SSTR-t imaging may become a useful tool for assessing post-MI local NP response and evaluating feedback to immunomodulatory therapy. But, why NPs need SSTR2 in context of MI is a question that requires separate research, and unfortunately, we cannot answer it within the framework of this study.

While all identified NPs were SSTR2 positive, only some of the Mos and MPs were SSTR2 positive. We hypothesize that some subsets of Mos and MPs express SSTR2 more than others. There is a fairly large diversity of Mo and MP subpopulations, which may differ significantly in their properties. Therefore, to better understand the mechanisms that determine the presence of SSTR2+ Mos and SSTR2+ MPs in the IC and BZ, it was necessary to phenotype these cells. However, this work did not involve the phenotyping of Mos and MPs, so this question remains for future studies.

We assume that in most cases the SSTR2 positive expression in blood vessels is caused by pericytes, in some cases by endothelial cells, but further studies of the co-expression of SSTR2 and type-specific cell markers by these cells are necessary. The literature has shown that pericytes and MPs are able to express SSTR2 in affected arteries in vasculitis of large vessels [33]. Moreover, it is known that pericytes play a key role in maintaining the function of the microvasculature and the remodeling of the vascular network, and are in close interaction with vascular endothelial, smooth muscle cells. The data from recent studies also indicate that pericytes are capable of acting as mesenchymal progenitor cells in cardiovascular organ regeneration [55]. The ability to non-invasively image activate pericytes has potential diagnostic and prognostic value. However, the distribution pattern of SSTR2+ pericytes and their role in post-MI regeneration are not fully understood, which requires further investigation.

At first glance, our results contradict one of the major previous works. According to the Tarkin et al., SSTR2 was not expressed by any of the following cells: Mos, T or B lymphocytes, natural killer cells, platelets, NPs, and endothelial cells [41]. However, our data demonstrate that NPs and Mos show clear positive expression of SSTR2 in addition to MPs. This discrepancy can be explained by differences in the methods (RT-PCR or IHC) and patient cohorts. Our study included patients with fatal MI; such a systemic catastrophe is associated with the generalized activation of the immune system. Apparently, NPs and Mos are activated in MI patients and exhibit higher SSTR2 expression. However, other potential causes cannot be ruled out. In addition, the authors performed an experiment to activate peripheral blood Mos with LPS, and showed that SSTR2 mRNA expression increased manifold [41]. Perhaps, NPs will also increase the expression of SSTR2 when we try to activate them experimentally.

The limitations of this work were the small sample of patients, and the identification of the SSTR2+ cell type was based on the characteristic light-optical morphology of the cells without the use of double IHC staining.

This pilot study provides initial insights into the morphology underlying SSTR-t imaging in MI, but many unanswered questions remain. Since octreotide, in addition to having a high affinity for SSTR2, has a moderate affinity for SSTR5, it is necessary to examine the cardiac expression of SSTR5 to see the whole picture. Further studies with a larger sample of patients, immunophenotyping of SSTR2-positive cells, and an analysis of correlations between clinical and morphological data are planned.

## 5. Conclusions

For the first time, we described the IHC distribution pattern of SSTR2-positive cells in the myocardium of MI patients and the control group.

SSTR2+ NPs and SSTR2+ Mos/MPs and SSTR2+ vessels were present in the myocardium of MI patients and the control group, but their number and distribution were significantly different.

During the inflammatory phase of MI in the human myocardium, the vast majority of SSTR2+ cells were located in the IC.

In the IC, SSTR2+ NPs were the predominant type of SSTR2+ cells; the number of SSTR2+ Mos/MPs in the IC was significantly lower than those of SSTR2+ NPs. SSTR2+ vessels were present, but their numbers were very low.

The RZ and the control myocardium did not differ in their number of SSTR2+ NPs and SSTR2+ vessels. Although the absolute number of SSTR2+ Mos/MPs in the RZ and in the control myocardium was very small, there were statistically significantly more SSTR2+ Mos/MPs in the control group than in the RZ.

In the IC, the median number of SSTR2+ NPs was 200 times higher than in the RZ, which may explain the selective uptake of SSTR-t radiotracers in the MI area.

There is a common pattern of SSTR2+ cell distribution in the myocardium of MI patients, but there is also variability in the number of SSTR2+ cells from patient to patient. Additional data may provide a basis for identifying different phenotypes of the local inflammatory response in MI patients.

## Figures and Tables

**Table 1:** Anamnestic characteristics of MI patients.

Patient Number	No. 1	No. 2	No. 3	No. 4	No. 5	No. 6	No. 7	All (n = 7)
Days from onset of MI to death	2	2	3	3	5	5	5	3 (2; 5)
Age, years	74	83	65	73	73	62	84	73 (65; 83)
Males, n (%)	M	F	M	F	M	M	F	4 (57)
Hypertension stage, n (%)	III	III	III	III	III	III	III	III–7 (100)
Type 2 diabetes, n (%)	yes		yes			yes		3 (43)
Dyslipidemia, n (%)	yes	yes	yes	yes	yes	yes	yes	7 (100)
Obesity, n (%)			yes	yes				2 (29)
History of COPD, n (%)	yes		yes			yes		3 (43)
Smoking, n (%)	yes		yes			yes		3 (43)
History of stroke, n (%)								0 (0)

**Note:** MI—myocardial infarction; M—male; F—female; COPD—chronic obstructive pulmonary disease. Data are presented as median (lower quartile; upper quartile) or number (%) of patients.

**Table 2:** Clinical and anamnestic characteristics of MI.

Patient Number	No. 1	No. 2	No. 3	No. 4	No. 5	No. 6	No. 7	All (n = 7)
Days from onset of MI to death	2	2	3	3	5	5	5	3 (2; 5)
STEMI, n (%)	yes	yes	yes	yes	yes	yes	yes	7 (100)
Localization of MI	Ant.W	Ant.W,Post.W	Ant.W, Post.W	Ant.W	Ant.W	Ant.W	Ant.W, Lat.W	Ant.W–7 (100) Post.W–2 (29) Lat.W–1 (14)
Recurrent MI, n (%)			yes			yes		2 (29)
History of HF, n (%)	yes		yes					2 (29)
GRACE, in-hospital death, % *	40	60	40	2	5	3	24	24 (3; 40)
Killip class *	4	4	4	1	1	4	3	Killip 1–2 (29) Killip 3–1 (14) Killip 4–4 (57)
LVEF (B), % *	21	43	29	60	37	32	41	37 (29; 43)
Total number of segments with violation of local contractility *	8	-	13	3	12	12	9	10.5 (8; 12)
Local contractility impairment index *	2	-	2.5	1.375	2.25	2.25	2.12	2.185 (2.0; 2.25)
Acute LV aneurysm *, n (%)					yes	yes	yes	3 (43)
IRA	LAD	CА	LAD	LAD	LAD	LAD	LAD	LAD—6 (86)
TIMI flow grade in IRA, before PCI → after PCI	0→3	0→3	0→3	0→0	0→2	0→3	0→3	0→3–5 (71) 0→2–1 (14) 0→0–1 (14)
Total ischemic time from the first ECG with ↑ST to reperfusion, min	165	183	355	2546	108	1409	68	183 (108; 1409)
Total ischemic time from the onset of pain to reperfusion, min	325	200	576	2582	573	4536 (>24 h)	151	573 (200; 2582)
Cause of death	CS	CS	CS	MR	RE-MI, CS	CS	MR	CS–5 (71) MR–2 (29)

**Note:** *—at the time of admission; MI—myocardial infarction; STEMI—ST-segment elevation myocardial infarction; Ant.W—anterior wall; Post.W—posterior wall; Lat.W—lateral wall; HF—heart failure; LV—left ventricle; LVEF—left ventricular ejection fraction; PCI—percutaneous coronary intervention; IRA—Infarct-related artery; LAD—left anterior descending artery; CA—circumflex artery; CS—cardiogenic shock; MR—myocardial rupture; RE-MI—reinfarction. Data are presented as median (lower quartile; upper quartile) or number (%) of patients.

**Table 3:** Laboratory parameters of MI patients.

Patient Number	No. 1	No. 2	No. 3	No. 4	No. 5	No. 6	No. 7	All (n = 7)
Days from onset of MI to death	2	2	3	3	5	5	5	3 (2; 5)
Hemoglobin, g/L *	100	104	161	144	126	150	145	144 (104; 150)
CBC. RBC, ×10^12^/L	4.61	4.06	5.41	5.46	4.3	4.47	5.13	4.61 (4.3; 5.41)
CBC. WBC, ×10^9^/L *	10.02	12.87	19.24	12.79	10.83	20.64	17.42	12.87 (10.83; 19.24)
CBC. Neutrophils, ×10^9^/L *	9.11	10.39	18.4	8.53	7.94	17.33	10.4	10.39 (8.53; 17.33)
CBC. Monocytes, ×10^9^/L *	0.56	0.48	0.57	1.28	1.37	1.19	1.49	1.19 (0.56; 1.37)
CBC. Lymphocytes, ×10^9^/L *	0.34	1.93	0.58	2.89	1.51	2.11	5.67	1.93 (0.58; 2.89)
CBC. Platelets, ×10^9^/L *	319	224	238	235	194	235	280	235 (224; 280)
Creatinine, µmol/l	121	120	163	66	80	73	118	118 (73; 121)
GFR, mL/min (Cockcroft-Gault formula)	53.6	29.7	67.8	116.5	74.0	99.7	24.8	67.79 (29.74; 99.7)
Fibrinogen, g/L *	3.00	-	4.94	4.80	5.80	3.99	4.80	4.8 (3.99; 4.94)
Troponin, ng/L * (N < 20.7)	+	3405.7	25,000	+	40,000	25,000	587.0	25,000 (3405.7; 25,000)
CPK, IU/L * (N < 171.0)	237	372	3016	251	1602	1185	175	372 (237; 1602)
CPK-MB, IU/L * (N < 25.0)	41	77	326	48	123	96	24	77 (41; 123)

**Note:** *—at the time of admission; MI—myocardial infarction; CBC—complete blood count; RBC—red blood cells; WBC—white blood cells; GFR—glomerular filtration rate; CPK—creatine phosphokinase. Data are presented as median (lower quartile; upper quartile) or number (%) of patients.

**Table 4:** Morphological data on the distribution of SSTR+ cells in the myocardium of MI patients and control group.

Patient Group		MI, Inflammatory Phase								Control Group		
Patient Number		No. 1	No. 2	No. 3	No. 4	No. 5	No. 6	No. 7	All (n = 7)	Con. No. 1	Con. No. 2	Con. No. 3
Days from onset of MI to death		2	2	3	3	5	5	5	3 (2; 5)			
**SSTR2+** **Mos/MPs**	IC	4.9	4.4	6.0	9.3	8.8	3.7	5.4	6 (4.9; 8.8)	0.8	1.0	0.5
	BZ	0.4	0.5	0.1	1.7	1.4	1.9	0.3	0.5 (0.3; 1.7)			
	RZ	0	0	0.2	0.1	0.7	0.4	0.4	0.2 (0; 0.4)			
	PSZ	-	-	1.7	-	-	1.8	-				
**SSTR2+** **NPs**	IC	126.8	112.3	47.9	248.6	169.8	116.4	31.0	116.4 (47.9; 169.8)	0	0.5	0.9
	BZ	9.2	8.1	0.8	14.8	3.0	2.4	0.3	3 (0.8; 9.2)			
	RZ	0.5	0.4	0.3	0.9	1.6	1.0	0.4	0.5 (0.4; 1)			
	PSZ	-	-	0.1	-	-	1.2	-				
**SSTR2+** **Vessels**	IC	0	0	0	0	0	0	0.4	0 (0; 0)	0	0	0
	BZ	0.1	0	0	0	0	0.5	0	0 (0; 0.1)			
	RZ	0	0.1	0	0	0	0	0.3	0 (0; 0.1)			
	PSZ	-	-	4.8	-	-	1.7	-				
Hemorrhagic impregnation		yes	yes	yes		yes		yes	5 (71)			
Scar				yes			yes		2 (29)			

**Note:** SSTR2—somatostatin receptor type 2; Mos/MPs—monocytes/macrophages; NPs—neutrophils; IC—infarct core; BZ—border zone; RZ—remote zone; PSZ—peri-scar zone. For SSTR2+ macrophages/monocytes and SSTR2+ neutrophils, numbers are given as the average number of cells per field of view; for SSTR2+ vessels, numbers are given as the average number of vessels per field of view. Data are presented as median (lower quartile; upper quartile) or number (%) of patients.

## Data Availability

The data obtained in this study are available upon request to the corresponding author. The data are not publicly available because the research is still in progress. However, data will be publicly available after the end of all studies connected to this preliminary phase.
